# *Fusobacterium nucleatum* promotes chemoresistance to 5-fluorouracil by upregulation of BIRC3 expression in colorectal cancer

**DOI:** 10.1186/s13046-018-0985-y

**Published:** 2019-01-10

**Authors:** Sheng Zhang, Yongzhi Yang, Wenhao Weng, Bomin Guo, Guoxiang Cai, Yanlei Ma, Sanjun Cai

**Affiliations:** 1 Department of Colorectal Surgery, Fudan University Shanghai Cancer Center, 270 Dong’an Road, Shanghai, 200032 China; 2 Department of Oncology, Shanghai Medical College, Fudan University, Shanghai, 200032 China; 3 Department of Clinical Laboratory, Yangpu Hospital, Tongji University School of Medicine, Shanghai, China; 4 Center for Translational Medicine, Yangpu Hospital, Tongji University School of Medicine, Shanghai, China; 5 Department of Surgery, Shanghai Jiao Tong University Affiliated Sixth People’s Hospital, Shanghai, China

**Keywords:** *Fusobacterium nucleatum*, BIRC3, 5-fluorouracil, Chemoresistance, Colorectal cancer

## Abstract

**Background:**

Emerging evidence suggests a potential relationship between gut microbiota and the host response to chemotherapeutic drugs including 5-fluorouracil (5-Fu). *Fusobacterium nucleatum (Fn)* has been linked to the initiation and progression of colorectal cancer (CRC). Unfortunately, little was known about the relationship between *Fn* infection and chemotherapeutic efficacy. Here, we investigate the potential relationship between *Fn* infection and chemotherapeutic efficacy of 5-Fu in CRC.

**Methods:**

Differentially expressed genes of CRC cell lines induced by *Fn* infection were analyzed based on a whole genome microarray analysis Then, we explored the relationship between upregulation of BIRC3 induced by *Fn* infection and chemoresistance to 5-Fu in vitro and in vivo. Furthermore, we dissected the mechanisms involved in *Fn*-induced BIRC3 expression. Finally, we investigated the clinical relevance of *Fn* infection, BIRC3 protein expression and chemoresistance to 5-Fu treatment in CRC patients.

**Results:**

BIRC3 was the most upregulated gene induced by *Fn* infection via the TLR4/NF-κB pathway in CRC cells; *Fn* infection reduced the chemosensitivity of CRC cells to 5-Fu through upregulation of BIRC3 in vitro and in vivo. High *Fn* abundance correlated with chemoresistance in advanced CRC patients who received standard 5-Fu-based adjuvant chemotherapy after radical surgery.

**Conclusions:**

Our evidence suggests that F*n* and BIRC3 may serve as promising therapeutic targets for reducing chemoresistance to 5-Fu treatment in advanced CRC.

**Electronic supplementary material:**

The online version of this article (10.1186/s13046-018-0985-y) contains supplementary material, which is available to authorized users.

## Background

Colorectal cancer (CRC) is the third most common cancer and the second leading cause of cancer-related death, accounting for 1.4 million new cases and 0.6 million deaths worldwide in 2012 [1]. In China, there were more than 0.37 million new cases of CRC that led to more than 0.19 million deaths in 2015 [2]. Recently, accumulating evidence has shown that intestinal bacteria dysbiosis is related to the initiation and development of CRC [3–5]. Moreover, the gut microbiota can also influence the efficacy of antitumor immunotherapy drugs [6–9] and have an impact on the treatment of CRC [10]. Interestingly, two studies have shown that bacterial metabolism can affect the host response to chemotherapeutic drugs including 5-fluorouracil (5-Fu) in *C. elegans* [11, 12]. *Fusobacterium nucleatum (Fn)*, an anaerobic bacterium parasitic in the oral cavity, is increasingly linked to CRC [13–18]. However, few studies have focused on the impact of *Fn* on the treatment of CRC.

The inhibitor of apoptosis proteins (IAPs) are characterized by the presence of baculoviral IAP repeat (BIR) domains which are important for the binding and inhibition of caspases [19–21]. They can promote the survival of tumor cells and induce chemoresistance [22]. Therefore, IAPs have attracted wide attention as potential targets for cancer therapy [23]. BIRC3 is a member of the IAP family that can inhibit apoptosis by directly inhibiting the caspase cascade [24, 25]. BIRC3 can also contribute to chemoresistance in malignancies including CRC [26]. Our previous study using microarray analysis showed that *Fn* can significantly induce BIRC3 expression in CRC cell lines. Based on this finding, we hypothesize that the significant upregulation of BIRC3 expression induced by *Fn* might be responsible for chemoresistance in CRC.

In this study, we demonstrate that *Fn* infection reduced the chemosensitivity of CRC cells to 5-Fu through upregulation of BIRC3 in vitro and in vivo, and high *Fn* abundance correlates with chemoresistance in advanced CRC patients who received standard 5-Fu-based adjuvant chemotherapy after radical surgery. Our evidence suggests that *Fn* and BIRC3 may serve as promising therapeutic targets for reducing chemoresistance to 5-Fu treatment in advanced CRC.

## Methods

### Bacteria strains and cell lines

*Fn* strain ATCC 25586 was purchased from American Type Culture Collection (ATCC) and grown in Columbia blood agar (Sigma, USA) in an anaerobic bag (Merier, France) at 37 °C as previously described [15]. HCT116, HT29 and 293 T cells were obtained from GeneChem and cultured in DMEM-F12(Gibco, USA) supplemented with 10% FBS (Gibco) and 1% penicillin and streptomycin (Beyotime, China) at 37 °C in a humidified 5% CO2 atmosphere. For *Fn* infection assay, cells were cultured in medium without antibiotics and incubated with *Fn* at a multiplicity of infection (MOI) of 100:1 as previously described [27].

### Patients and specimens

A total of 94 patients diagnosed with advanced CRC were included in this study. All the patients received standard 5-Fu-based adjuvant chemotherapy after radical surgery in Fudan University Cancer Center from 2007 to 2017. None of them received preoperative treatment. Ninety-four formalin-fixed paraffin-embedded (FFPE) CRC tissues were obtained from the pathological archives. Prognostic information was collected by the medical record system and telephone follow-up. The median follow-up time was 38.5 months, ranging from 7 to 132 months. During the follow-up period, 45 patients (47.8%) suffered from recurrence of the disease. Clinicopathological data of the patients are summarized in Table 1. Written informed consent was obtained from the patients, and the study was approved by the Ethics Committee of the hospital.

**Table 1:** Clinicopathological characteristics of CRCs in according to Fn abundance or recurrence status

Characteristics	All cases	*Fn*-low/negative	*Fn*-high	P value	Non-recurrence	Recurrence	*P* value
	(*n* = 94)	(*n* = 73)	(*n* = 21)		(*n* = 49)	(*n* = 45)	
Age				0.849			0.646
≥ 60	42(44.7%)	33 (45.2%)	9 (42.9%)		23 (46.9%)	19 (42.2%)	
<60	52 (55.3%)	40 (54.8%)	12(57.1%)		26 (53.1%)	26(57.8%)	
Sex				0.936			0.323
Female	41 (43.6%)	32 (43.8%)	9 (42.9%)		19 (38.8%)	22 (48.9%)	
Male	53 (56.4%)	41 (56.2%)	12 (57.1%)		30 (61.2%)	23 (51.1%)	
Tumor location				0.051			0.079
Rectum	54 (57.4%)	42 (57.5%)	12 (57.1%)		23 (46.9%)	31 (68.9%)	
Distal colon	23 (24.5%)	21 (28.8%)	2 (9.5%)		16 (32.7%)	7 (15.6%)	
Proximal colon	17 (18.1%)	10 (13.7%)	7 (33.3%)		10 (20.4%)	7 (15.6%)	
Tumor size				0.156			0.131
>4 cm	41 (43.6%)	29 (39.7%)	12(57.1%)		25 (51.0%)	16 (35.6%)	
≤ 4 cm	53 (56.4%)	44 (60.3%)	9 (42.9%)		24 (49.0%)	29 (64.4%)	
Differentiation				0.840			0.573
Well/Moderate	75 (79.8%)	59 (80.8%)	16 (76.2%)		38 (77.6%)	37 (82.2%)	
Poor	19 (20.2%)	14 (19.2%)	5 (23.8%)		11 (22.4%)	8 (17.8%)	
Vascular invasion				0.874			0.003
Positive	30 (31.9%)	23 (31.5%)	7 (33.3%)		9 (18.4%)	21 (46.7%)	
Negative	64 (68.1%)	50 (68.5%)	14 (66.7%)		40 (81.6%)	24 (53.3%)	
Neural invasion				0.808			0.029
Positive	22 (23.4%)	18 (24.7%)	4 (19.0%)		7 (14.3%)	15(33.3%)	
Negative	72 (76.6%)	55 (75.3%)	17 (81.0%)		42 (85.7%)	30 (6.7%)	
TNM stage				0.130			0.153
II	45 (47.9%)	38 (52.1%)	7 (33.3%)		20 (40.8%)	25 (33.3%)	
III	49 (52.1%)	35 (47.9%)	14 (66.7%)		29 (59.2%)	20 (66.7%)	
BIRC3 staining							0.008
Positive	36 (38.3%)	21 (28.8%)	15 (71.4%)	0.000	25 (51.0%)	11 (71.4%)	
Negative	58 (61.7%)	52 (71.2%)	6 (28.6%)		24 (49.0%)	34 (28.6%)	
TLR4 staining							0.036
Positive	50 (53.2%)	35 (47.9%)	15 (71.4%)	0.057	21 (42.9%)	29 (64.4%)	
Negative	44 (46.8%)	38 (52.1%)	6 (28.6%)		28 (57.1%)	16 (35.6%)	
*Fn* abundance							0.014
*Fn*-low/negative	73 (77.7%)				43(83.6%)	30 (71.1%)	
*Fn*-high	21 (22.2%)				6 (16.4%)	15 (28.9%)	

Pearson Chi-square test

### Quantitative real-time PCR

For detection of *Fn* abundance, genomic DNA (gDNA) was extracted from FFPE tissues with QIAamp DNA FFPE Tissue Kit (QIAGEN, Germany). The abundance of *Fn* was determined by detecting the 16S gene using qPCR. Each 10-μL reaction contained 80 ng of gDNA, 0.4 mM each primer and 1× final concentration of SYBR Green PCR Master Mix (Thermo Fisher Scientific, USA). Amplification was performed using the ABI Step One Plus Real-Time PCR System (Applied Biosystems, USA) under the following reaction conditions: 10 min at 95 °C, followed by 40 cycles of 95 °C for 15 s and at 60 °C for 1 min. The gene prostaglandin transporter (PGT) was used as the internal reference as previously described [13]. For detection of target gene expression, total RNA was isolated using TRIzol reagent (Invitrogen, USA), and 1 mg of total RNA was reverse transcribed using a reverse transcription kit (Promega, USA). Complementary DNA (cDNA) was amplified and quantified on the ABI Step One Plus Real-Time PCR System (Applied Biosystems) using SYBR Green (Takara, China) under the following reaction conditions: 30 s at 95 °C and 40 cycles of 5 s at 95 °C and 30 s at 60 °C. Q-PCR was performed in triplicate for each sample, and the 2^-ΔΔCt^ method was used to assess the relative expression levels of target genes as previously described [28]. GAPDH served as an internal reference gene and all the primer sequences are given in Additional file 1: Table S1.

### Microarray analysis

Total RNA was isolated from HCT116 cells infected with or without *Fn* for 24 h. Then, the probes were prepared by labeling the mRNA with Cy5-dUTP (for the control group) or with Cy3-dUTP (for the infected group) through reverse transcription. The labeled cDNA probes were hybridized to a Human Whole Genome Microarray (Zhuolibiotech, China) and scanned with an Axon 4000 scanner (Molecular Devices, Sunnyvale, CA, USA). Bioinformatics analysis was performed. The list of differentially expressed genes was filtered for fold changes > 2.0, and those genes were selected for further investigation.

### Cell proliferation assay and drug cytotoxicity assay

The percentage of viable cells was determined by Cell Counting Kit-8 (CCK8; Yeasen, China) assay under different treatment conditions. Briefly, cells were seeded in 96-well plates with 100 μL of culture medium. Ten microliters of CCK8 solution was added to each well at the indicated time points and incubated at 37 °C for 2 h. The reaction product was assessed by measuring the optical absorbance at 450 nm using a spectrophotometer (Thermo Fisher Scientific, USA).

### Western blot analysis

Total cellular protein was extracted and separated by 10% SDS-PAGE then transferred to polyvinylidene fluoride (PVDF) membranes. Membranes were blocked with 5% BSA in TBST for 1 h at room temperature and then incubated with the primary antibodies overnight at 4 °C, followed by incubation with secondary antibodies for 2 h at room temperature. The signals were detected using an enhanced chemiluminescence reagent (Millipore, USA). The information for all antibodies used is listed in Additional file 2: Table S2.

### Immunohistochemical staining and immunofluorescence staining

For immunohistochemistry (IHC), an EnVision+ Dual Link Kit (Dako, USA) was used according to the manufacturer’s instructions. The staining process was performed as previously described [29]. All sample slides were scored separately by two pathologists according to the distribution, intensity and percentage of positive cells [30]. Briefly, the staining intensity was scored as 0 (negative), 1 (weak), 2 (medium) or 3 (strong). The extent of staining was scored as 0 (< 5%), 1 (5–25%), 2 (26–50%), 3 (51–75%) or 4 (> 75%). The scores of intensity and extent were multiplied to generate the immunoreactivity score. Final staining scores 0–4 and 5–12 were considered negative and positive, respectively.

*Fn*-induced NF-κB activation was detected using an NF-κB Activation Nuclear Translocation Assay Kit (Beyotime, China) according to the manufacturer’s instructions [31]. Briefly, cells were fixed and washed followed by incubation with blocking buffer for 1 h. Then, cells were incubated with the primary antibody against NF-κB P65 overnight in a humidified chamber at 4 °C before a Cy3-conjugated secondary antibody was applied for 1 h at room temperature. Finally, nuclei of cells were stained with DAPI for 5 min. Images were captured by laser confocal microscopy at an excitation wavelength of 350 nm for DAPI (blue) and 540 nm for Cy3 (red), and merged using software.

### Apoptosis detection

The cell apoptosis ratio was determined by flow cytometric analysis with an Annexin V FITC/PI double stain assay (BD Biosciences, USA) according to the manufacturer’s instructions. Levels of apoptosis in the xenograft tumor tissues were determined by terminal deoxynucleotidyl transferase-mediated dUTP nick end labeling (TUNEL) technology using an Apoptosis Assay Kit (Beyotime, China).

### Small interfering RNA (siRNA) silencing in vitro

SiRNAs were purchased from GenePharma (Shanghai, China). Transfection of siRNA was performed using Lipofectamine 3000 (Invitrogen, USA) according to the manufacturer’s protocol and nonspecific siRNAs were used as negative controls. The sequences of siRNA used in this study are shown in Additional file 1: Table S1.

### Chromatin immunoprecipitation assay (ChIP)

ChIP assays were carried out using a ChIP Assay Kit (Beyotime, China) following by the manufacturer’s protocol. Briefly, cells were treated with 1% formaldehyde to cross-link DNA and proteins. Afterwards, the cell lysates were sonicated to generate chromatin fragments of 200–300 bp and immunoprecipitated with an anti-P65 antibody or IgG as a control. The region 5 kb upstream of BIRC3 gene was set as a negative control. The precipitated chromatin DNA was recovered and measured by qPCR. The percent input for each experiment was converted to fold change relative to an untreated control. Average fold changes from 3 independent experiments were then plotted. Primer sequences for the ChIP-qPCR assay are listed in Additional file 1: Table S1.

### Dual luciferase reporter assay

The NF-κB P65 binding motif in the promoter region of BIRC3 was predicted by JASPAR (http://jaspar.genereg.net/). Two putative binding sites with the highest score were selected. Wild-type (WT) or mutated putative NF-κB P65 binding sites in the BIRC3 promoter region were amplified and cloned into the pGL3-Promoter vector (Additional file 3: Figure S1). 293 T cells were transfected with WT or mutated BIRC3 promoter construct and pcDNA-P65 or pcDNA-NC vector (GeneChem, China) using Lipofectamine 3000 (Invitrogen, USA). Luciferase activity was determined using a Dual Luciferase Reporter Assay Kit (Promega, USA) according to the manufacturer’s protocol.

### Mouse model

For the xenograft experiments, 5-week-old male BALB/c nude mice (Chinese Academy of Sciences, Shanghai, China) were housed in laminar flow cabinets under specific pathogen-free conditions. To establish the CRC xenograft model., HCT116 (5 × 10^6^ cells/0.5 ml) cells were subcutaneously injected into the right flank of the mice. One week after subcutaneous inoculation, mice were randomly divided into 4 groups for different sets of experiments (*n* = 5). *Fn* and siRNA were administered by multipoint intratumor injection every 3 days for a total of five injections using an in vivo transfection reagent (Entranster™-in vivo, Engreen, China) according to the instructions [32]. 5-Fu (Absin, China) was administered by an intraperitoneal injection of 5 mg/kg per mouse, twice per week for 2 weeks. Tumor volume (V) was calculated using the following formula: V = (a × b^2^)/2, where a and b represent the longer and shorter tumor diameters, respectively. After the 2-week treatment, all mice were sacrificed and the tumors were collected. The samples were fixed with formalin for IHC, TUNEL assay and hematoxylin and eosin (H&E) staining. The animal study was approved by the Committee on Animals Handling of Fudan University Shanghai Cancer Center.

### Statistical analysis

Statistical analyses were performed using GraphPad Prism software 7.0 (GraphPad Software, La Jolla, CA). Data are presented as the means ± standard deviation. Unpaired Student t test or nonparametric Mann-Whitney test was applied to assess the statistical significance between groups with different treatment. Correlations were calculated according to Pearson Chi-square test All statistical tests were two-sided and *p* < 0.05 was considered statistically significant.

## Results

### *Fn* infection significantly induces expression of BIRC3 in CRC cell lines

**Fig. 1:**
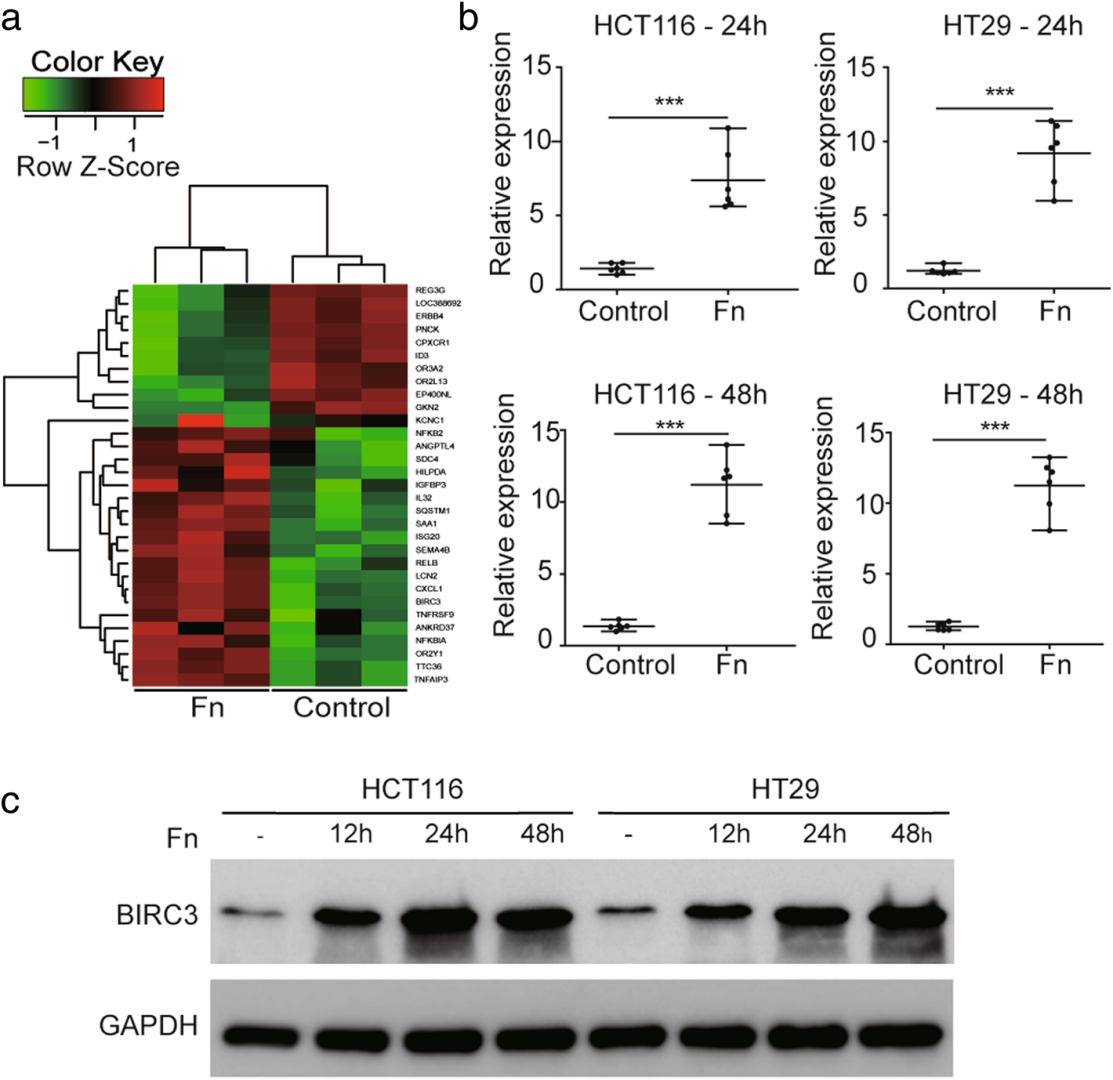
BIRC3 expression is upregulated by *Fn* infection in CRC cell lines. **a** Hierarchical clustering of the differentially expressed gene heatmap between HCT116 cells and *Fn*-infected HCT116 cells using Microarray analysis. These genes were affected by changes > 2-fold with *p* values below 0.05. BIRC3 was the gene with the highest expression (12-fold increase) induced by *Fn* infection (*n* = 3). **b** The relative mRNA expression of the BIRC3 gene determined by qPCR in CRC cell lines infected with or without *Fn* for 24 h and 48 h respectively. Data are shown as individual values with the median and range from three independent experiments performed in triplicate (*n* = 6) (***p < 0.001 by unpaired Student t test). **c** Representative Western blot for BIRC3 protein and GAPDH protein extracted from CRC cells infected with *Fn* for 0 h, 12 h, 24 h and 48 h

To assess the global effect of *Fn* infection in vitro, human CRC HCT116 cells were incubated with or without *Fn* for 24 h. Whole genome microarray analysis was performed and differentially expressed genes were analyzed. The result showed that BIRC3 was the gene with the highest expression (12-fold increase) induced by *Fn* infection compared with the control groups (Fig. 1a). To validate the upregulation of BIRC3 in CRC cell lines infected with *Fn*, qPCR and Western blot analysis were performed. The results showed that the mRNA levels of BIRC3 in HCT116 and HT29 cells were significantly upregulated by *Fn* infection for 24 h and 48 h respectively (Fig. 1b). Western blot analysis also showed that *Fn* infection increased the expression of BIRC3protein at different time intervals (Fig. 1c). As BIRC3 is a member of the IAP family [33], we also examined the protein levels of the other members of this family including BIRC2 (cIAP1), BIRC4 (XIAP) and BIRC5 (Survivin). The results showed that the protein levels of these genes was not affected by *Fn* infection (data not shown).

### *Fn* induces chemoresistance of CRC cells to 5-Fu via upregulation of BIRC3 in vitro

**Fig. 2:**
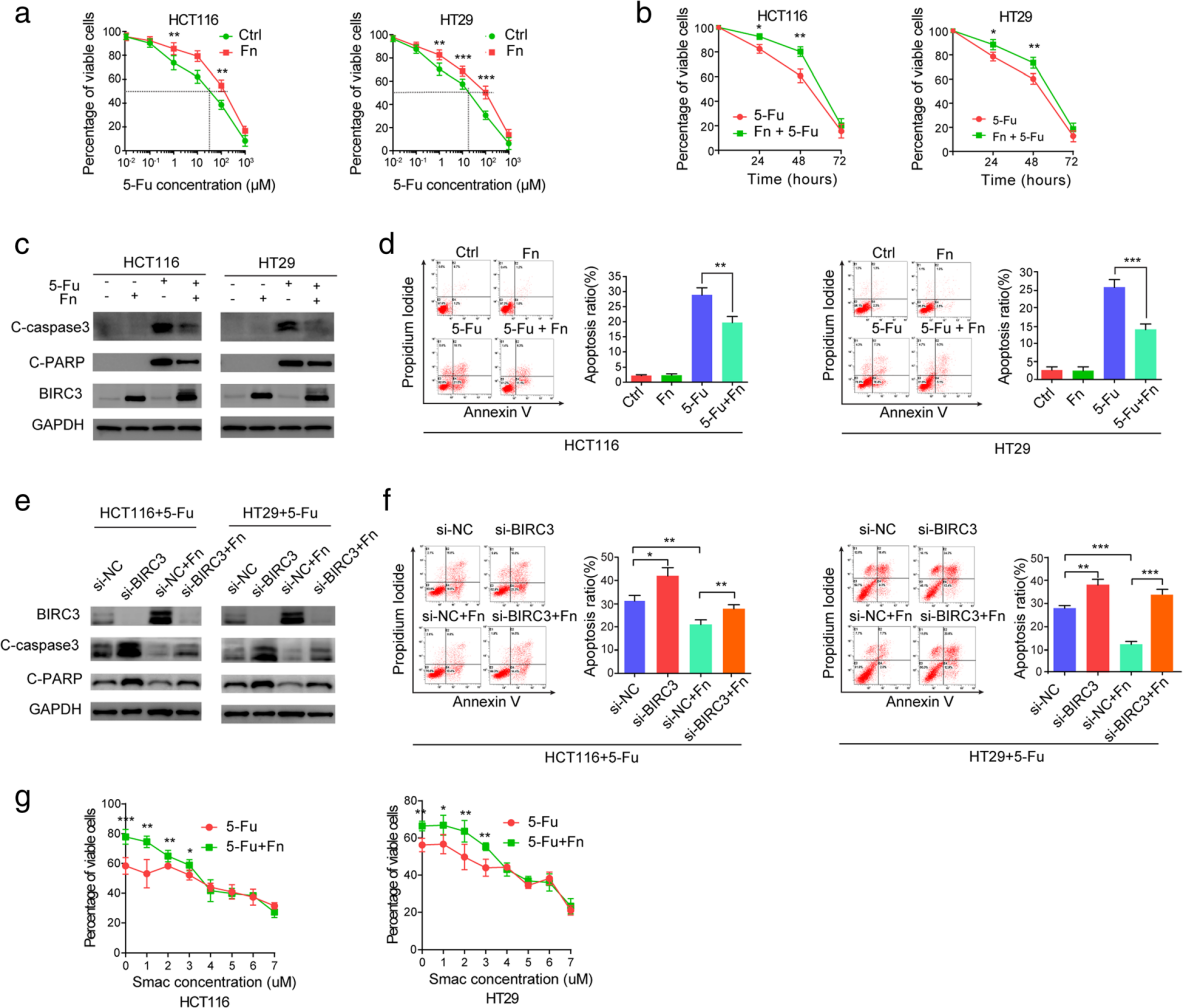
*Fn* induces chemoresistance of CRC cells to 5-Fu via upregulation of BIRC3 in vitro. **a** Cells were incubated with or without *Fn* and then exposed to serial dilutions of 5-Fu for 48 h. Cell viability was determined by CCK8 assay. Data are presented as the percentage of viable cells (mean ± s.d.). The IC_50_ of 5-Fu in HCT116 and HT29 cells was approximately 30 μM and 20 μM respectively, as demonstrated by the dotted line. **b** Cells were incubated with or without *Fn* and then exposed to IC_50_ 5-Fu. Cell viability was assessed by CCK8 assay at 0 h, 24 h, 48 h, and 72 h. Data are presented as the percentage of viable cells (mean ± s.d.). **c-d** Cells were incubated with or without *Fn* in the presence or absence of IC_50_ 5-Fu for 48 h. Cleaved of caspase-3 (C-caspase-3), cleaved of PARP (C-PARP) and BIRC3 were detected by Western blot (**c**). Apoptosis was detected by flow cytometry. Data are presented as the ratio of apoptotic cells (mean ± s.d.). Representative quadrantal diagrams (left) and the corresponding histogram (right) from three independent experiments are shown (**d**). **e-f** Cells were transfected with specific siRNA for 24 h and then incubated with or without *Fn* in culture medium with IC_50_ 5-Fu for an additional 48 h. Protein levels of BIRC3, C-caspase-3, and C-PARP were measured by Western blot (**e**). Apoptosis ration was detected with flow cytometry. Data are presented as the ratio of apoptotic cells (mean ± s.d.). Representative quadrantal diagrams (left) and the corresponding histogram (right) from three independent experiments are shown (**f**). **g** Cells were incubated with or without *Fn* and then exposed to gradient concentrations of a SMAC mimetic in the culture medium with IC_50_ 5-Fu for 48 h. Cell viability was determined by CCK8 assay and the data are plotted. Data are presented as the percentage of viable cells (means±s.d.) from three independent experiments (*p < 0.05; **p < 0.01; ***p < 0.001 by unpaired Student t test)

Since BIRC3 is a member of the IAP family that inhibits apoptosis by directly inhibiting caspase cascade [25], and can contribute to chemoresistance in malignancies including CRC [26, 34], we speculated that the upregulation of BIRC3 expression induced by *Fn* infection may lead to chemoresistance in CRC. To test this hypothesis, we initially determined whether *Fn* infection decreased cytotoxicity induced by 5-Fu in CRC cells. The results showed that *Fn* decreased cytotoxicity induced by 5-Fu in HCT116 and HT29 cells (Fig. 2a). We also found that the 50% inhibitory concentration (IC_50_) of 5-Fu in HCT116 and HT29 cells was approximately 30 μM and 20 μM, respectively (Fig. 2a). Based on this result, cells were incubated with or without *Fn* and then exposed to IC_50_ 5-Fu and cell viability was assessed at the indicated time. Consistent with previous results, *Fn* infection significantly reduced cytotoxicity induced by 5-Fu treatment for 24 h or 48 h(Fig. 2b). To address whether *Fn* infection protects CRC cells from 5-Fu-mediated apoptosis, we cocultured HCT116 and HT29 cells with or without *Fn* in the presence or absence of IC_50_ 5-Fu for 48 h. Western blotting showed that the cleavage of caspase3 (C-caspase3) and cleavage of PARP (C-PARP) was induced by 5-Fu, while this effect was attenuated by *Fn* infection (Fig. 2c). Flow cytometry analysis with Annexin V FITC/PI double stain assay was performed to assess the apoptosis ratio of each group. The results showed that 5-Fu-induced apoptosis was crippled by *Fn* infection (Fig. 2d). Taken together, these results suggest that *Fn* infection can reduce the chemosensitivity of CRC cells to 5-Fu in vitro.

To confirm the role of BIRC3 in the *Fn*-induced chemoresistance to 5-Fu, BIRC3 gene was silenced in CRC cells using specific siRNA. Cells were incubated with or without *Fn* and then exposed to IC_50_ 5-Fu for 48 h. Western blotting showed that *Fn* infection crippled the level of C-caspase3 and C-PARP induced by 5-Fu, while this effect was abolished by silencing the BIRC3 gene in the cells (Fig. 2e). Under the same conditions, the apoptosis ratio of each group was detected with flow cytometry. We also observed that silencing of BIRC3 abrogated *Fn*-induced chemoresistance of the cells in response to 5-Fu (Fig. 2f). Furthermore, a small molecule antagonist of BIRC3 named SMAC mimetic (SM-406, Selleck, China) was used to antagonize the effect of BIRC3 [35, 36]. The results showed that the chemoresistance effect mediated by *Fn* gradually weakened with increasing of SMAC mimetic concentration in the cells (Fig. 2g). Altogether, these data strongly suggest that BIRC3 plays an essential role in the *Fn*-induced chemoresistance of CRC cells in response to 5-Fu in vitro.

### The TLR4/NF-κB pathway regulates BIRC3 expression in CRC cells cocultured with *Fn*

**Fig. 3:**
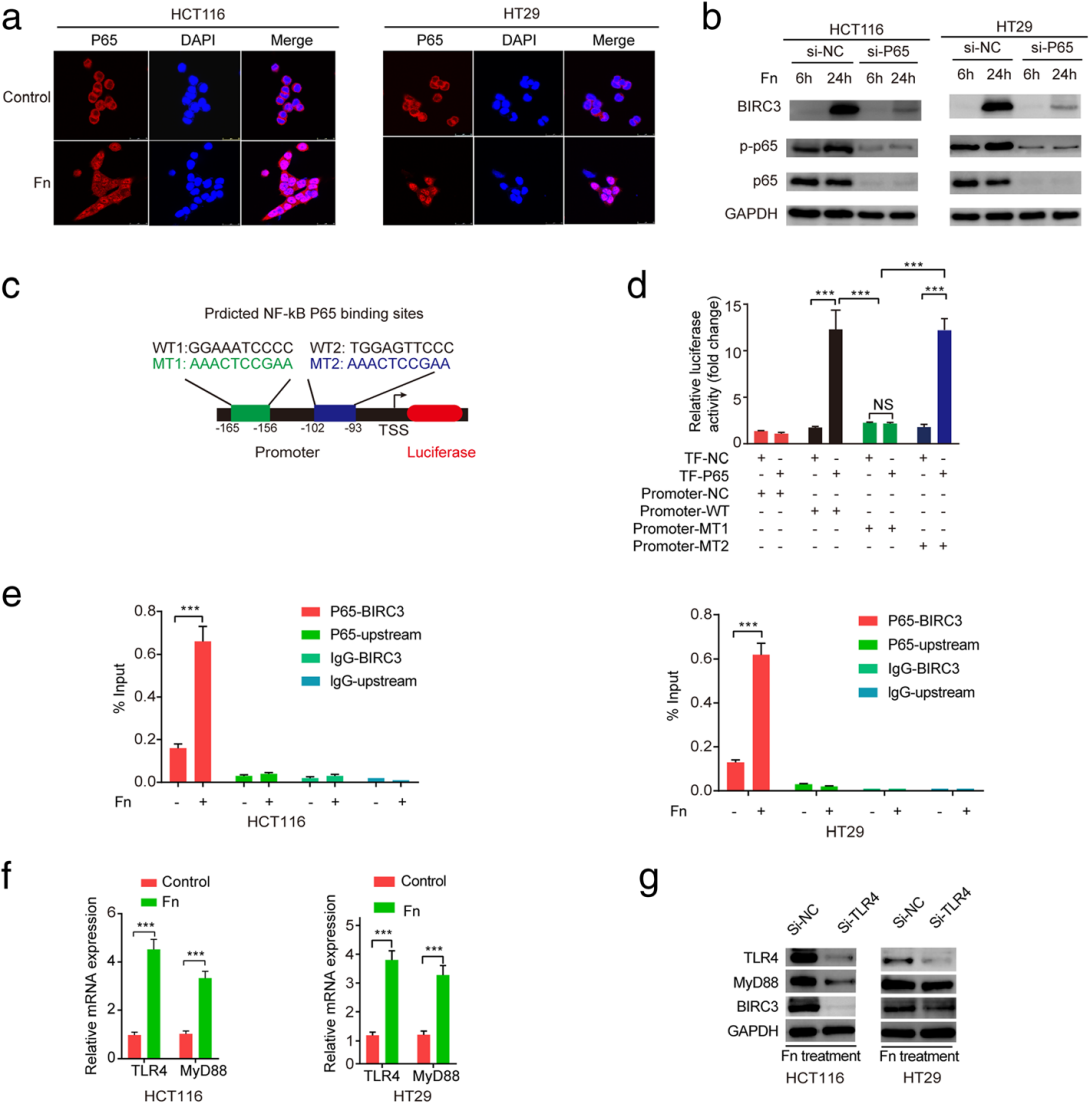
*Fn* induces BIRC3 expression through TLR4/NF-kb pathway. **a** Cells were cocultured with or without *Fn* for 2 h followed by incubation with an antibody against NF-κB P65 and a Cy3 fluorescein-conjugated secondary antibody, and nuclei were stained with DAPI. **b** Cells were transfected with specific siRNA (si-P65 or si-NC) for 24 h followed by cocultured with *Fn* for an additional 6 h or 24 h. Protein levels of BIRC3, p-P65, and P65 were measured by Western blot. **c** Schematic representation of the location of two predicted NF-κB P65 binding motifs in the BIRC3 promoter region. WT: wild-type; MT1: mutant type 1; MT2: mutant type 2. **d** pGL3-Promoter reporter plasmids (NC, WT, MT1, MT2) and pcDNA3.1-TF-cDNA vectors (TF-NC, TF-P65) were generated. Dual luciferase reporter assay were carried out according to different combinations. TF: transcription factor; NS: nonsignificant. **e** Cells were cocultured with or without *Fn* for 2 h. Then, ChIP assays were carried out with an anti-P65 antibody or rabbit IgG. qPCR was then carried out for the BIRC3 promoter region or the region 5 kb upstream of BIRC3 gene. **f** Cells were co-cultured with or without *Fn* for 24 h. The relative mRNA level of TLR4 and MyD88 was detected by qPCR. **g** Cells were transfected with specific siRNA (si-TLR4 or si-NC) for 24 h followed by co-cultured with *Fn* for an additional 48 h. Protein levels of TLR4, MyD88, and BIRC3 were measured by Western blot. (***p < 0.001 by unpaired Student’s t-test)

It was previously reported that *Fn* infection can activate the TLR4/NF-κB pathway in CRC cells [17, 37] and bioinformatics analysis of the microarray data also supported this conclusion. In addition, NF-κB activation induces the expression of a variety of target genes including BIRC3 [38, 39]. Based on this evidence, we speculated that the TLR4/NF-κB pathway may be involved in the *Fn*-induced upregulation of BIRC3. First, we observed NF-κB P65 nuclear translocation when the cells were incubated with *Fn* for 2 h. (Fig. 3a). Western blot analysis showed that *Fn* infection contributed to NF-κB P65 activation and upregulation of BIRC3, while silencing of NF-κB P65 abrogated the *Fn*-mediated upregulation of BIRC3 in the cells. (Fig. 3b). To examine the direct interaction between NF-κB P65 and the BIRC3 promoter region, a dual luciferase reporter assay was performed. The result showed that cotransfection of plasmids containing transcription factor P65 (TF-P65) and the wild-type (WT) pGL3-Promoter vector resulted in a significant increase in promoter activity compared to the transcription factor-negative (TF-NC) group (Fig. 3d), suggesting that NF-κB P65 positively regulates the activity of the BIRC3 promoter. To further explore the potential NF-κB P65 binding sites on the BIRC3 promoter region, two mutant type (MT) pGL3-Promoter vectors were generated (Fig. 3c and Additional file 3: Figure S1) and independently cotransfected with TF-P65 or TF-NC. We observed that cotransfection of the pGL3-MT2-Promoter plasmid resulted in the same effect on promoter activity as that with the pGL3-WT-Promoter plasmid, while this effect was not observed with the cotransfection of the pGL3-MT1-Promoter plasmid (Fig. 3d). Therefore, we may infer that the binding site is located 156 to 165 bp upstream of the transcription start site (TSS) (Fig. 3c). Furthermore, ChIP assays were performed for P65 in CRC cells. We found that recruitment of P65 to the BIRC3 promoter region was enhanced by *Fn* infection in the cells (Fig. 3e). To investigate whether TLR4 and MyD88 are involved in the *Fn*-mediated upregulation of BIRC3, we first confirmed that *Fn* infection can upregulate the levels of TLR4 and MyD88 transcripts in the cells. (Fig. 3f). Next, cells were transfected with specific siRNA (si-TLR4 or si-NC) for 24 h followed by coculture with *Fn* for additional 48 h. Western blotting analysis showed that silencing of TLR4 crippled *Fn*-mediated upregulation of BIRC3 in the cells.(Fig. 3g). Collectively, these results support the hypothesis that the *Fn* induces BIRC3 expression through TLR4/NF-κB pathway in CRC cells.

### *Fn* induces chemoresistance of CRC cells in response to 5-Fu in vivo

**Fig. 4:**
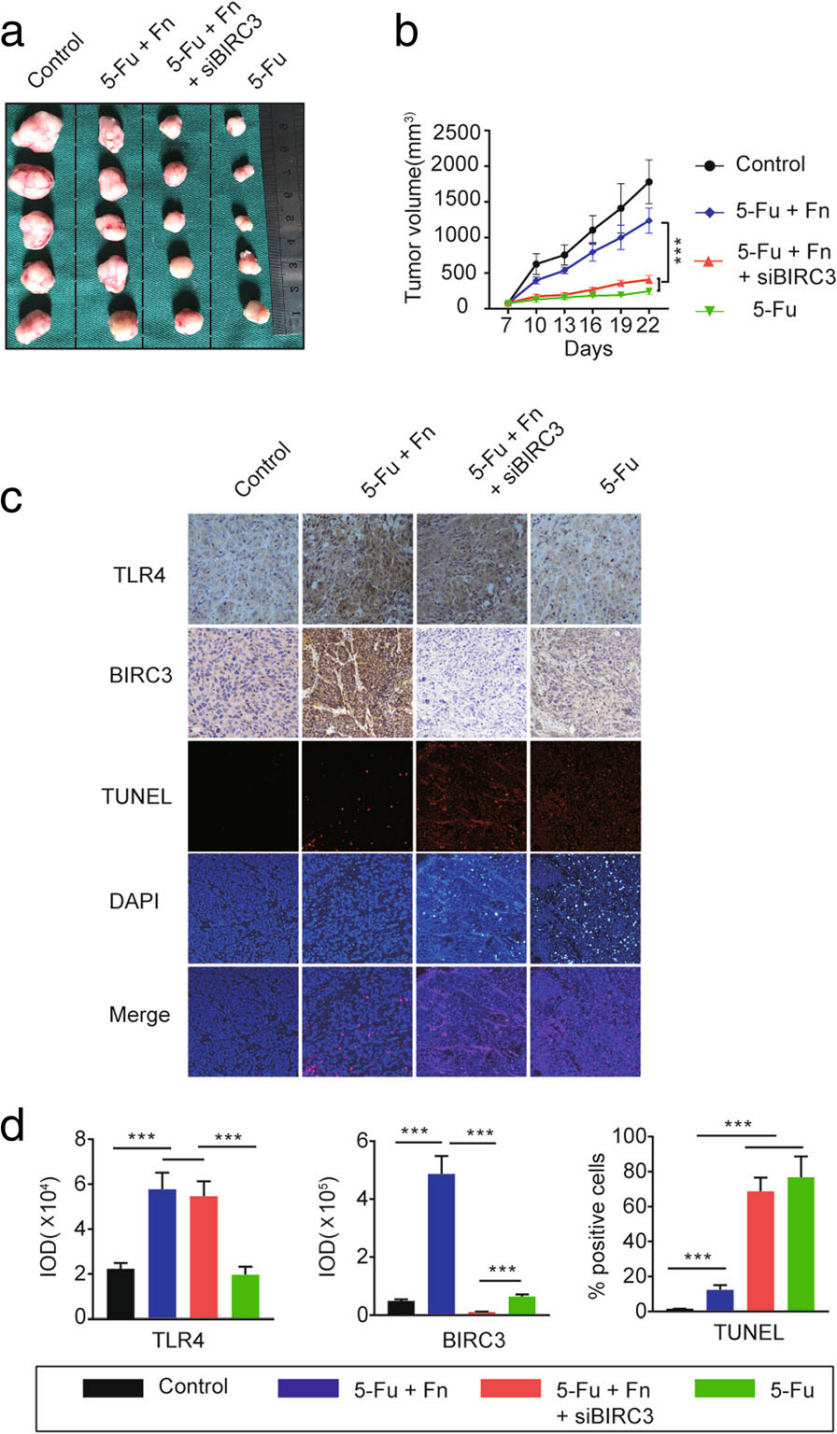
*Fn* induces chemoresistance of CRC cells in response to 5-Fu in vivo. **a** After the 2-week treatment, mice under different conditions were sacrificed and the tumors were collected and photographed. **b** Tumor sizes were measured every 3 days and tumor growth curves were established. Data are presented as tumor volume (mean ± s.d.; ***p < 0.001 by nonparametric Mann-Whitney test). **c** Immunohistochemical staining was carried out to detect the proteins expression levels of TLR4 and BIRC3, and a TUNEL assay was performed to detect tumor cell apoptosis in xenograft tumor tissues. **d** Integrated optical density (IOD) values were obtained using Image-Pro Plus software (Media Cybernetics, Rockville, MD, USA) and used to quantify the protein expression levels of TLR4 and BIRC3. Data are presented as IOD values. (mean ± s.d.; ***p < 0.001 by nonparametric Mann-Whitney test). As for TUNEL assay, data are presented as the percentage of TUNEL- positive cells per high-power field of the various tumor sections. (mean ± s.d.; ***p < 0.001 by nonparametric Mann-Whitney test)

A subcutaneous xenograft model was used to further explore whether *Fn* induces chemoresistance in CRC cells and to determine the role of BIRC3 in this effect in vivo. Four groups were designed according to the purpose of this experiment. The results showed that tumor growth was significantly decreased by 5-Fu treatment while this effect was crippled by *Fn* infection in vivo (Fig. 4a-b). Interestingly, silencing of BIRC3 partially attenuated the *Fn*-mediated chemoresistance of tumors in response to 5-Fu in vivo (Fig. 4a-b). These findings were further confirmed by TUNEL assay (Fig. 4c-d). To explore the relationship between *Fn* infection and the expression level of TLR4 and BIRC3 in vivo, IHC was carried out in tumor sections. We observed that *Fn* infection significantly upregulated the expression levels of TLR4 and BIRC3 (Fig. 4c-d). In conclusion, these data indicate that BIRC3 plays an essential role in the *Fn*-mediated chemoresistance of CRC cells in response to 5-Fu in vivo.

### High *Fn* abundance correlates with poor recurrence-free survival (RFS) in advanced CRC

**Fig. 5:**
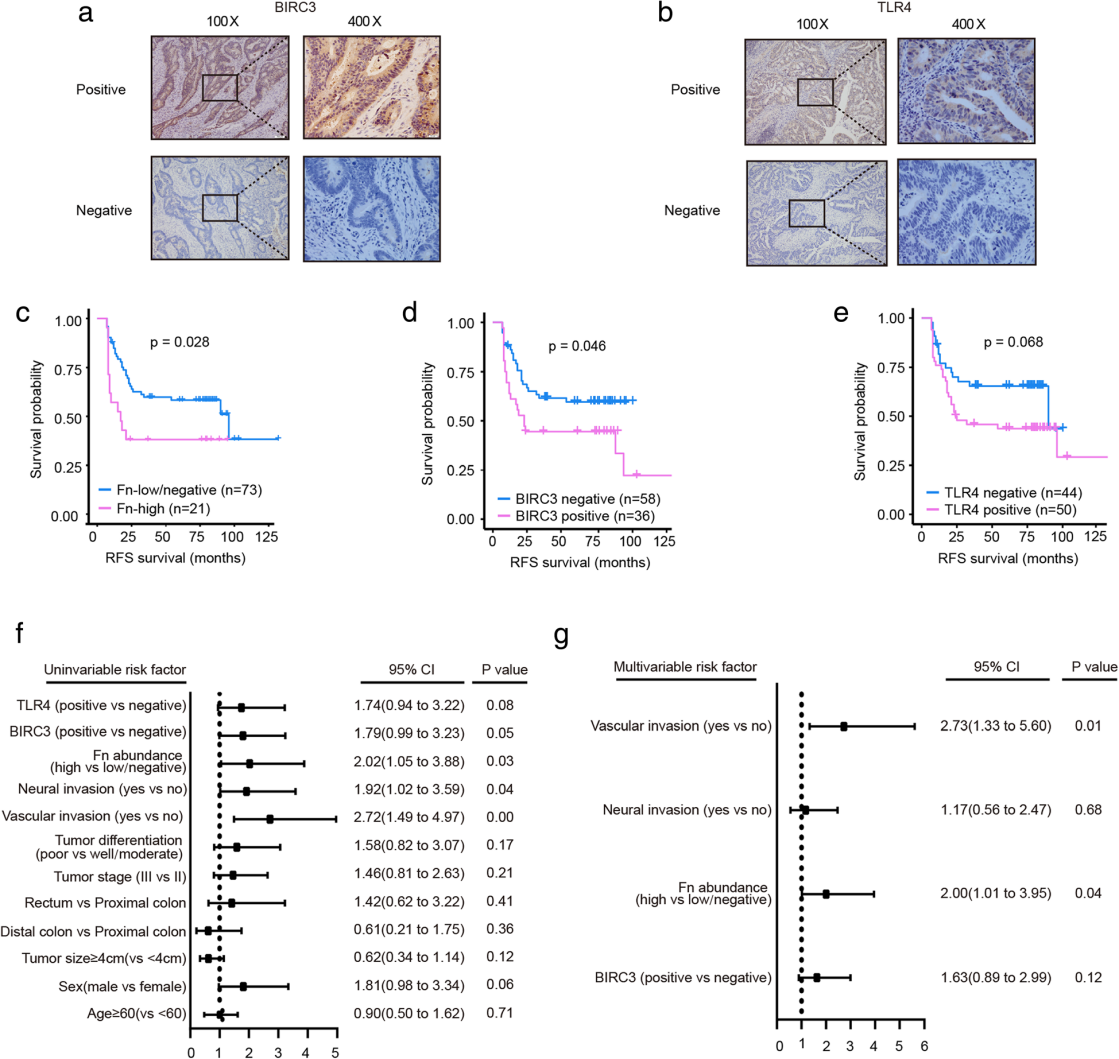
High *Fn* abundance correlates with poor RFS in advanced CRC**. a-b** Representative IHC images of BIRC3 (**a**) and TLR4 (**b**) proteins in FFEP CRC tissues**. c-e** RFS was compared between patients with high and low/negative abundances of *Fn* (**c**), between the BIRC3 -positive group and BIRC3-negative group (**d**), and between the TLR4-positive group and TLR4-negative group (**e**) using Kaplan-Meier analysis and the log-rank test. **f-g** Univariate (**f**) and multivariable (**g**) analyses were performed in the 94 patients included. CI: confidence interval

To investigate whether *Fn* infection and its downstream target genes can affect the efficacy of 5-Fu-based chemotherapy in CRC patients, we initially detected the *Fn* abundance and BIRC3 and TLR4 expression levels in FFPE CRC tissues from patients who were pathologically diagnosed with advanced CRC and received standard 5-Fu-based adjuvant chemotherapy after radical surgery. The distribution of *Fn* abundance is shown in Additional file 4: Figure S2. We set a cut-off value of 0.1 (2^-ΔCt^) for *Fn* abundance and divided the patients into the *Fn*-low/negative group and the *Fn*-high group as previously described [40]. Based on this division, we identified 22.3% of patients as having a high amount of *Fn.* The expression level of TLR4 and BIRC3 in FFEP tissues were determined by IHC assay. and divided into positive or negative status. Representative IHC images of BIRC3 and TLR4 proteins in FFEP CRC tissues were shown in Fig. 5a (BIRC3) and Fig. 5b (TLR4). As shown in Table 1, BIRC3 expression level was positively correlated with *Fn* abundance (71.4% in the *Fn*-high group vs. 28.8% in the *Fn*-low/negative group; *p* < 0.001). We also observed a trend of higher TLR4 expression in the *Fn*-high group (71.4%) than in the *Fn*-low/negative group (47.9%), although there was no statistical significance (*p* = 0.057). In line with our previous in vitro findings, these results suggested that *Fn* infection and its downstream target genes TLR4 and BIRC3 are clinically relevant in CRC patients.

Moreover, high *Fn* abundance (*p* = 0.014), high levels of TLR4 (*p* = 0.036) and BIRC3 (*p* = 0.008) proteins were more likely detected in patients with recurrence, compared with patients without recurrence.(Table 1). In addition, vascular invasion (*p* = 0.003) and neural invasion (*p* = 0.029) positively correlated with recurrence status (Table 1). Kaplan-Meier analysis showed that high amount of *Fn* (Fig. 5c, *p* = 0.028) and high level of BIRC3 expression (Fig. 5d, *p* = 0.046) correlated with poor RFS. However, the level of TLR4 expression was independent of RFS (Fig. 5e, *p* = 0.068). Univariate Cox regression analyses for RFS were performed and the results showed that the risk of recurrence was associated with high *Fn* abundance (HR = 2.02; 95% CI = 1.05 to 3.88; *p* = 0.03), positive vascular invasion (HR = 2.72; 95% CI = 1.49 to 4.97; *p* = 0.00) and positive neural invasion (HR = 1.92; 95% CI = 1.02 to 3.59; *p* = 0.04) (Fig. 5f). In the multivariate Cox regression analysis for RFS, the risk of recurrence was correlated with high amount of *Fn* (HR = 2.00; 95% CI = 1.01 to 3.95; *p* = 0.04) and positive vascular invasion (HR = 2.73; 95% CI = 1.33 to 5.60; *p* = 0.01) (Fig. 5g). Taken together, these results demonstrate that high *Fn* abundance is an independent risk factor for recurrence in advanced CRC patients who received standard 5-Fu-based adjuvant chemotherapy after radical surgery, and further support that upregulation of BIRC3 expression induced by *Fn* might be responsible for chemoresistance in CRC.

## Discussion

Fluorouracil-based adjuvant chemotherapy is a standard treatment for advanced CRC patients [41]. 5-Fu chemoresistance is a major challenge and the prognosis for CRC patients can be very poor due to recurrence of disease [42]. However, the potential mechanisms involved in the resistance to chemotherapeutic drugs including 5-Fu are not fully understood. It has been reported that drug absorption disorder, changes in drug targets, activation of DNA repair pathways, inhibition of apoptosis, and the tumor microenvironment are involved in drug resistance [43, 44]. The mechanism of 5-Fu resistance is associated with the activity of enzymes involved in the modulation of 5-Fu metabolism, such as thymidylate synthase, thymidine phosphorylase and dihydropyrimidine dehydrogenase [41]. Microsatellite instability status also correlates with chemosensitivity to 5-Fu [45]. Interestingly, increasing evidence suggests that gut microbiota may affect the efficacy of antitumor drugs [10, 46]. In this study, we initially found that BIRC3 was the most upregulated gene induced by *Fn* infection in CRC cell lines. Moreover, BIRC3 is a member of the IAP family that inhibits apoptosis by directly inhibiting the caspase cascade [24, 25], which has potential to aid in the treatment of CRC [19]. As inhibition of apoptosis is an important mechanism of drug resistance [47], we speculated that the upregulation of BIRC3 induced by *Fn* may be associated with chemoresistance. It is well acknowledged that 5-Fu-based chemotherapy is the principal postoperative treatment for advanced CRC [48]. Therefore, we hypothesized that *Fn* infection and upregulation of BIRC3 may be involved in resistance to 5-Fu. To test this hypothesis, we first demonstrated that *Fn* infection reduced the chemosensitivity of CRC cells to 5-Fu in vitro and in vivo. Next, we confirmed that this biological effect depends on the upregulation of BIRC3. Furthermore, we dissected the mechanisms involved in *Fn*-induced BIRC3 expression, which showed that the TLR4/NF-κB pathway regulates BIRC3 expression in CRC cells that are cocultured with *Fn*. Finally, to correspond to the aforementioned findings, we investigated the clinical significance of *Fn* infection and its downstream target genes. The results showed that a high amount of *Fn* was an independent risk factor for recurrence in advanced CRC patients.

Many studies have reported that overexpression of BIRC3 is associated with chemoresistance in the treatment of malignancies. Karasawa et al. found that BIRC3-positive patients had a shorter disease-free survival after 5-Fu-based chemotherapy [26] and suggested that BIRC3 can serve as a therapeutic target in CRC and other malignancies [49]. Krajewska et al. found that elevated expression of BIRC3 significantly correlated with shorter overall survival in early-stage CRC [50]. In addition, overexpression of BIRC3 significantly correlated with resistance to several chemotherapeutic drugs, including 5-Fu, in pancreatic cancer cells [34]. Upregulation of BIRC3 induced by IL-1β results in chemoresistance to doxorubicin in breast cancer cells [51]. Consistent with these findings, our results showed that a high level of BIRC3 induced by *Fn* infection contributed to chemoresistance to 5-Fu in vitro, and correlated with poor RFS in CRC patients. A recent study reported that *Fn* can mediate chemoresistance by autophagy pathway activation in CRC [52]. In line with this, our study observed similar phenomena elucidated with different mechanism. Another study found that *Fn* can migrate with CRC cells to metastatic sites and that CRC patients may benefit from eradication of *Fn* with antibiotic treatment [53]. Therefore, we may infer that *Fn* as a part of metastatic tumor plays a role in the chemoresistance to 5-Fu treatment and contributes to disease recurrence. However, there are some limitations in this research. First, this study just focuses on *Fn* enriched in tumor tissues and the indirect effect of *Fn* on 5-Fu. It is still unknown that whether *Fn* inhabiting in intestinal lumen of CRC patients can directly affect the efficacy of 5-Fu. Second, subcutaneous xenograft cannot simulate intestinal microenvironment very well. So, further study is needed to investigate the direct interactions between *Fn* and chemotherapeutic drugs.

## Conclusions

**Fig. 6:**
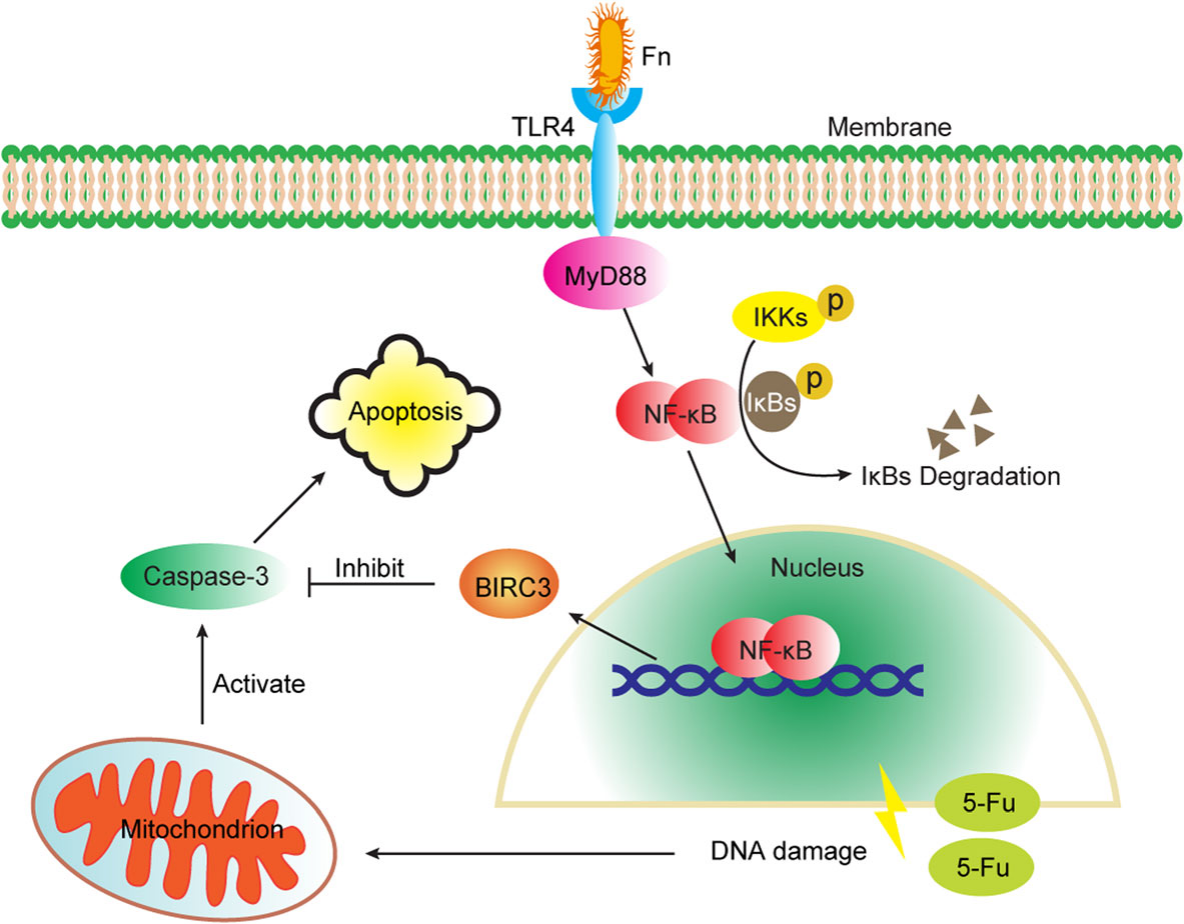
Schematic diagram of the mechanism by which *Fn* protects CRC cells from 5-Fu-mediated apoptosis via upregulation of BIRC3 expression

In conclusion, our study initially found that *Fn* infection reduces the chemosensitivity of CRC cells to 5-Fu through the upregulation of BIRC3, and that the TLR4/NF-κB pathway regulates BIRC3 expression in CRC cells cocultured with *Fn* (Fig. 6). Targeting *Fn* infection and BIRC3 may be promising in the treatment of advanced CRC patients who received 5-Fu-based chemotherapy after radical surgery, and may optimize the current treatment strategy of CRC.

## Additional files

Additional file 1: **Table S1.** Primer sequences of qPCR and ChIP-qPCR and siRNA sequences used in the study. (XLSX 10 kb)

Additional file 2: **Table S2.** Information of antibody used in the study. (XLSX 9 kb)

Additional file 3: **Figure S1.** The peak map of DNA sequencing about the constructions of wild-type and mutant BIRC3 promoters. (PDF 712 kb)

Additional file 4: **Figure S2.** Distribution of *Fn* abundance in CRC patients (*n* = 94)**.** The relative abundance of *Fn* in FFPE CRC tissues was determined by 2^-ΔCt^. The patients were ranked according to abundance of *Fn*. A cut-off value of 0.1 was set to distinguish the high *Fn* abundance group (*n* = 21) from the low/negative *Fn* abundance group(*n* = 73). (TIF 508 kb)

## Abbreviations

5-Fu: 5-fluorouracil
ATCC: American Type Culture Collection
CCK8: Cell Counting Kit-8
ChIP: Chromatin immunoprecipitation assay
CRC: Colorectal cancer
FFPE: Formalin-fixed paraffin-embedded
*Fn*: *Fusobacterium nucleatum*
gDNA: Genomic DNA
IAP: Inhibitor of apoptosis protein
IC_50_: 50% inhibitory concentration
IHC: Immunohistochemistry
MOI: Multiplicity of infection
MT: Mutant type
PGT: Gene prostaglandin transporter
qPCR: Quantitative real-time polymerase chain reaction
RFS: Recurrence-free survival
siRNA: Small interfering RNA
TF-NC: Transcription factor-negative control
TF-P65: Transcription factor-P65
TSS: Transcription start site
TUNEL: Terminal deoxynucleotidyl transferase-mediated dUTP nick end labeling
WT: Wild-type
