# Supplementary material for: Fusobacterium nucleatum promotes chemoresistance to 5-fluorouracil by upregulation of BIRC3 expression in colorectal cancer
Source: J Exp Clin Cancer Res. 2019 Jan 10;38:14. doi: 10.1186/s13046-018-0985-y (PMC6327560; doi:10.1186/s13046-018-0985-y)
Supplement: Supplementary file 3 — Figure S1. The peak map of DNA sequencing about the constructions of wild-type and mutant BIRC3 promoters. (PDF 712 kb) [file 13046_2018_985_MOESM3_ESM.pdf]

GGAAATCCCC

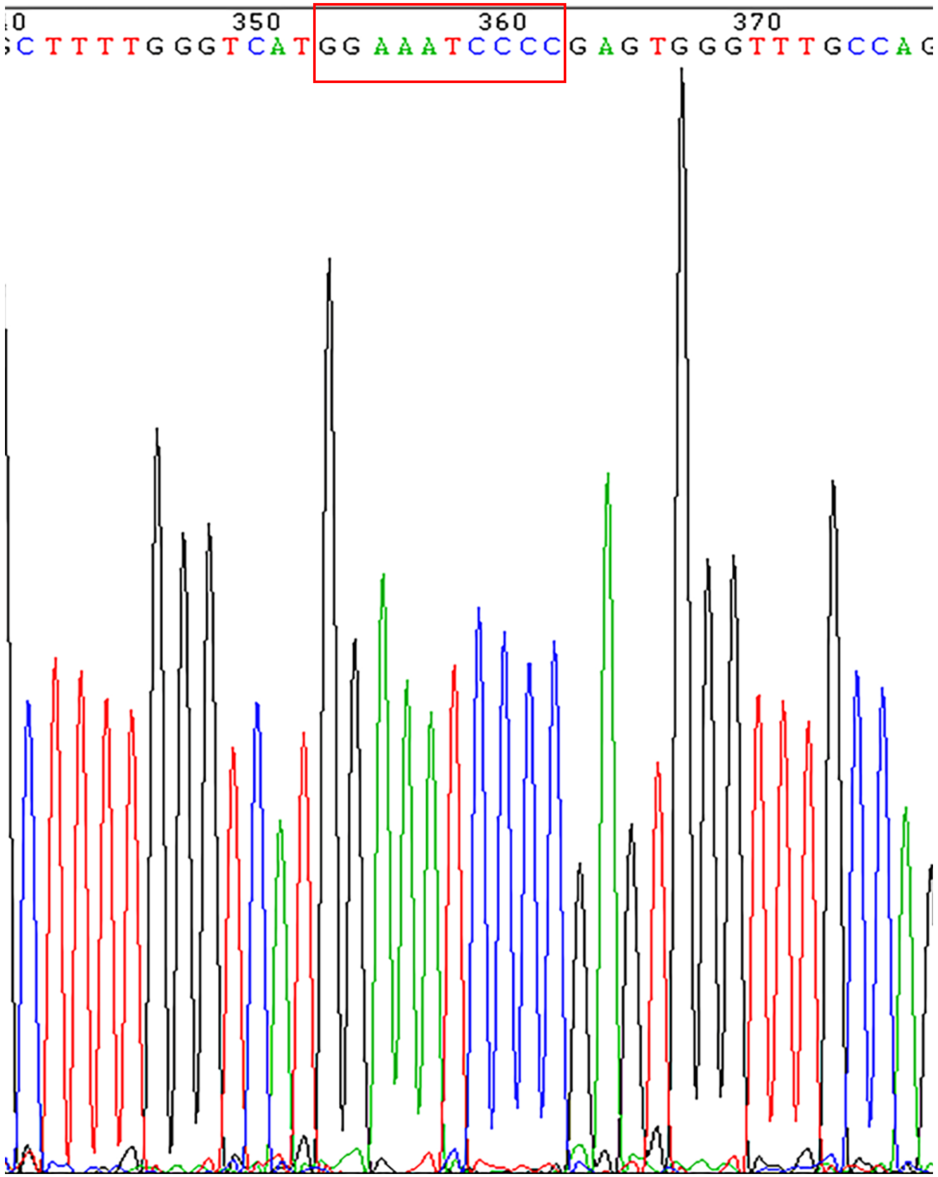

Wild type 1

AAACTCCGAA

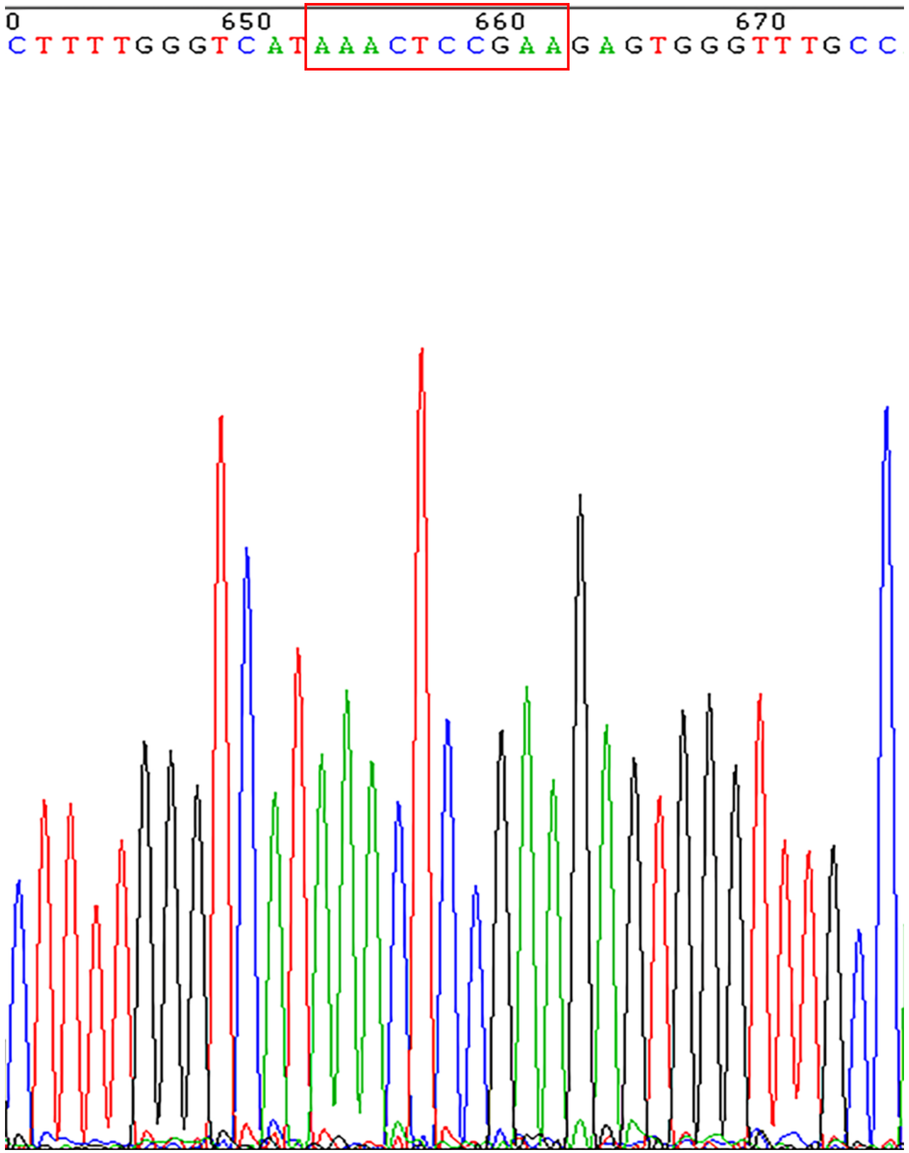

Mutant 1

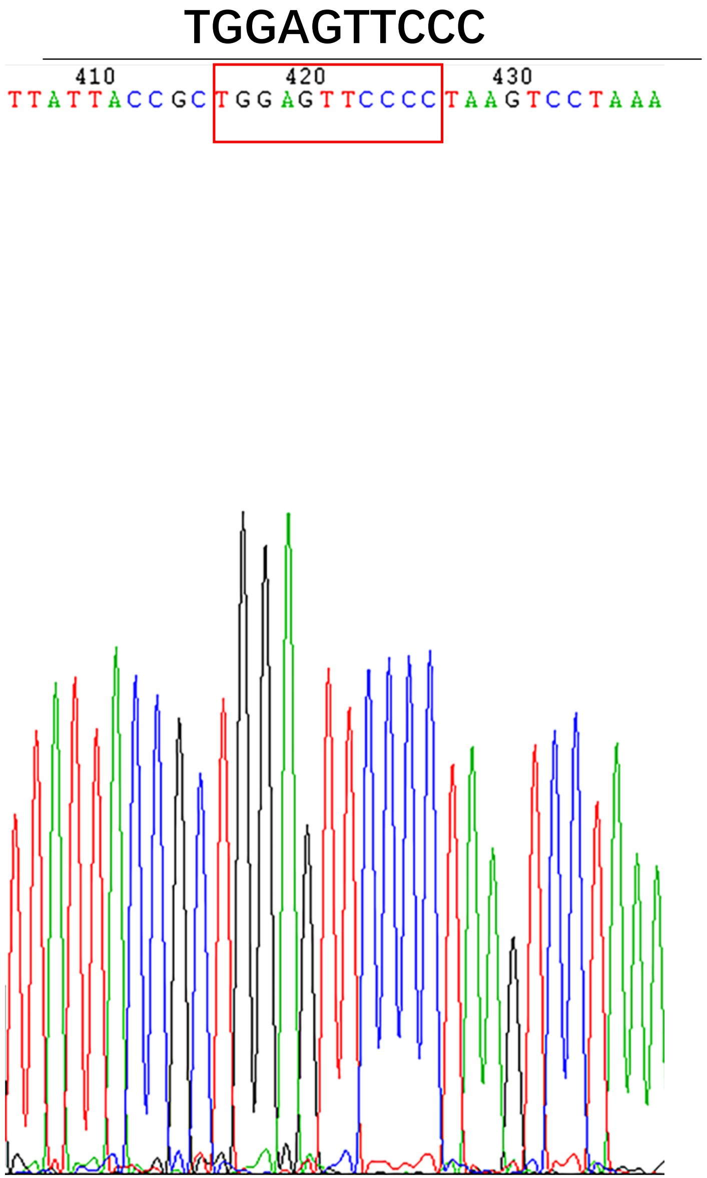

Wild type 2

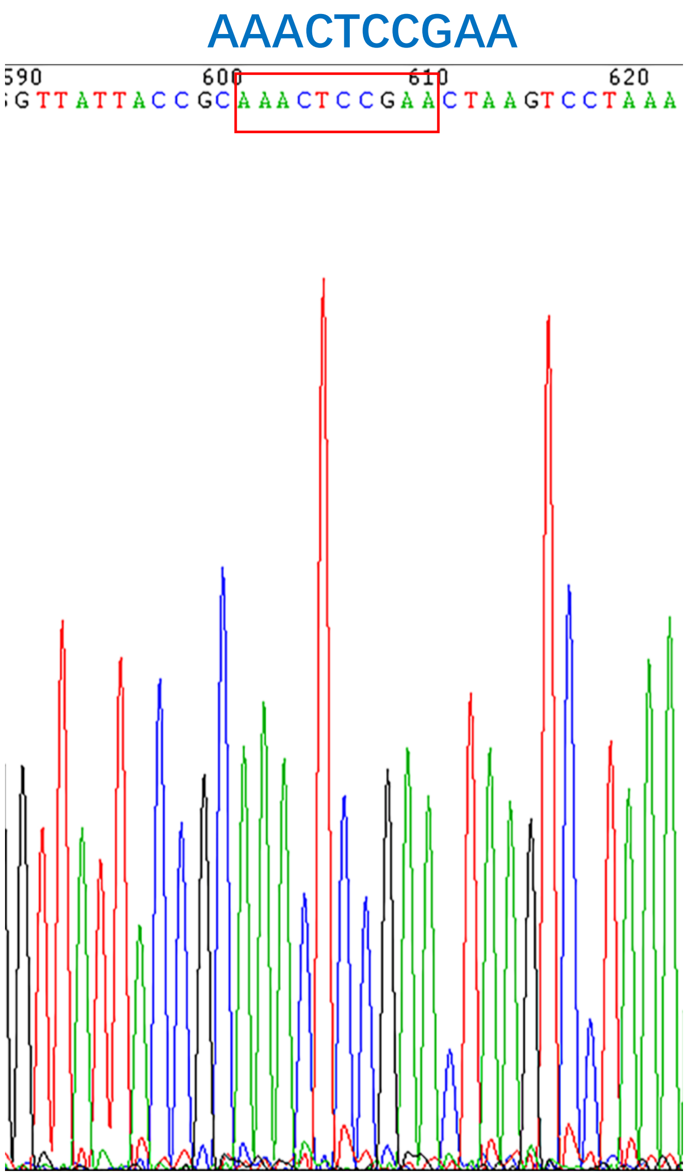

Mutant 2
