# Supplementary figures and images for: Fusobacterium nucleatum promotes chemoresistance to 5-fluorouracil by upregulation of BIRC3 expression in colorectal cancer
Source: J Exp Clin Cancer Res. 2019 Jan 10;38:14. doi: 10.1186/s13046-018-0985-y (PMC6327560; doi:10.1186/s13046-018-0985-y)

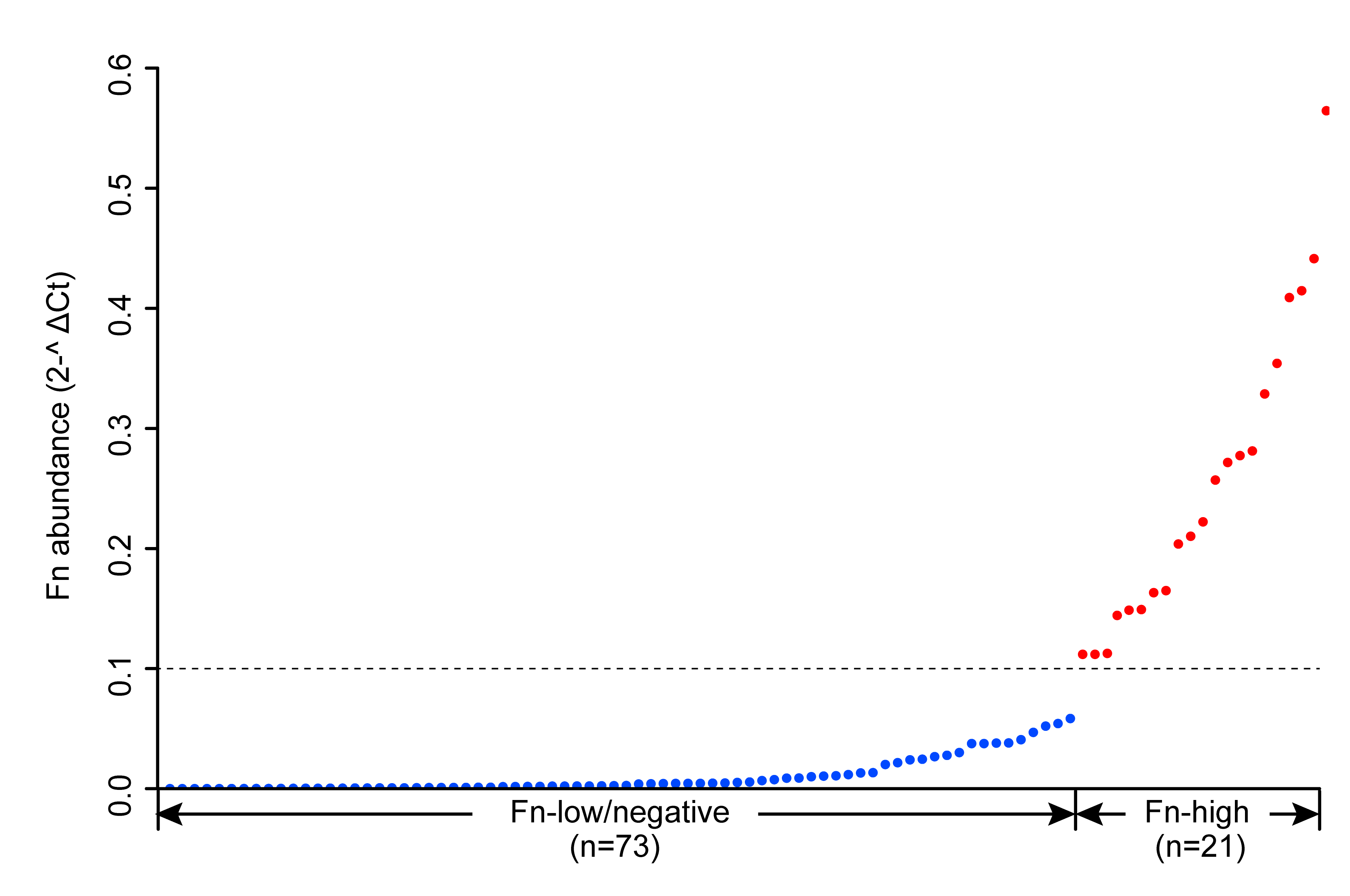

Supplement: Supplementary file 4 — Figure S2. Distribution of Fn abundance in CRC patients (n = 94). The relative abundance of Fn in FFPE CRC tissues was determined by 2-ΔCt. The patients were ranked according to abundance of Fn. A cut-off value of 0.1 was set to distinguish the high Fn abundance group (n = 21) from the low/negative Fn abundance group(n = 73). (TIF 508 kb) [file 13046_2018_985_MOESM4_ESM.tif]
